# Industry 4.0 Readiness Calculation—Transitional Strategy Definition by Decision Support Systems

**DOI:** 10.3390/s22031185

**Published:** 2022-02-04

**Authors:** Maja Trstenjak, Tihomir Opetuk, Hrvoje Cajner, Miro Hegedić

**Affiliations:** Faculty of Mechanical Engineering and Naval Architecture, University of Zagreb, Ivana Lucica 5, 10 000 Zagreb, Croatia; tihomir.opetuk@fsb.hr (T.O.); hrvoje.cajner@fsb.hr (H.C.); miro.hegedic@fsb.hr (M.H.)

**Keywords:** Industry 4.0, process planning, readiness factor, maturity, digitization, strategy, analytic hierarchy process, decision support

## Abstract

The digitization of the manufacturing industry, 10 years after the introduction of the Industry 4.0 concept, is still one of the most demanding tasks for the companies, especially for SMEs. As one of the biggest barriers in new business model implementation, the lack of strategy and workforce skills is frequently mentioned in the literature. The high level of investments it requires and the perception of high risks with unclear future benefits can be avoided with readiness factor calculation. This paper presents a novel model for readiness factor calculation, oriented to process planning and based on decision support systems. The model enables the definition of the optimal strategic plan for the digitization with the use of decision support systems (analytic hierarchy process) and through the use of statistical methods implemented within the model it minimizes the influence of human subjectivity and quantification of qualitative criteria. This innovative approach enables the understanding of the transition process to new technology-enabled business models, in this case oriented towards process planning. The useability and reliability of the model is proven in a case study of a metal machining company.

## 1. Introduction

Process planning is one of the key stages in the product lifecycle. The accuracy of the decision-making level at this point impacts the future cost of manufacturing and the product quality [1]. As any other phase of manufacturing and design, process planning is now required for certain changes and the complete digitization in terms of the Industry 4.0 concept, as the current trends on the market suggest, in order to retain the level of competitiveness [2]. Process planning in the literature is most commonly understood as part of the product design phase or the beginning stage of the manufacturing planning and scheduling. Therefore, conceptual views on digital process planning using the Industry 4.0 principles are rarely present in the literature. The transition to Industry 4.0 requires many changes in the current system. In order to avoid additional costs, minimize risks, and increase the value of future benefits, an optimal strategy plan has to be defined [3]. This can be provided by the readiness factor calculation, as the starting point which gives the company an overview of the current position compared to the ideal model/work environment by Industry 4.0 principles. The readiness factor calculation should be a quantitative and objective evaluation of Industry 4.0 features (elements) in a certain company [4].

Therefore, the purpose of this paper is to define the novel approach to the objective readiness factor calculation, specialized in the process planning phase which results in an optimal strategic plan of digitization/transformation of the company.

The general evaluation results of the current state in the work environment are based on the evaluation of various technical, organizational, and social criteria where each has a certain importance/weight. Hence the goal of this research is to define the novel readiness factor calculation model through decision support system principles, which would give an answer to the following research questions:

RQ1: How to define an optimal strategy plan of the digital transformation of process planning?

RQ2: Can the use of decision support systems in readiness factor calculation process improve the accuracy of the transitional strategy?

RQ3: How can the impact of human subjectivity in the readiness factor calculation for the digital transformation be minimalized?

Currently, the most advanced traditional process planning method or tool is CAPP (computer-aided process planning), but the digital environment requires a more complex system that would be linked to as many subjects in the value chain as possible, define the process plan with all required data automatically with the possibility of continuous self-optimization in real time [5].

### Process Planning in Industry 4.0

Process planning, as a step between the product design and physical manufacturing, tends to be automatized. In the context of Industry 4.0, it requires the specific digitalization which implies the connection with other phases of the product lifecycle, but also the continuous optimization by the results of the big data analytics with the flexible characteristics. The idea of the process planning automatization (automatic definition of process plan) begins with the CAPP (computer-aided process planning) concept—the use of computer technology to aid in the process planning of a product, which is a link between the CAD and CAM modules [6]. One of the most important parts of the process planning automation is the automatic recognition of the geometrical features of the product [7,8,9]. However, CAPP methods that optimize plans in a linear manner have not been able to satisfy the need for flexible planning, so new dynamic systems would need to explore all possible combinations of production processes. One of the presented solutions is the use of the genetic algorithm [10].

Process planning in the Industry 4.0 environment is very closely related to production scheduling which defines the process plan in the real time in which the machine and resources availability influence the selection and order of the manufacturing operations and its regimes in real time. The digital process planning presented in the [11] is based on CAPP, which is upgraded with special control and optimization algorithms based on the advanced predictive analytics and decision support systems. Apart from the standard data of product design, the process plan is generated by available scheduling and availability data of machines and resources in real time. To improve final product quality, the feedback data from every phase of a product’s lifecycle is available to process planner in the advanced ERP system and influences the continuous improvement of the process planning algorithms. 

Krolikowski and Krawczyk [12] recognized the need for optimization and digitization in production of complex products that contain geometrical features which are hard to achieved with conventional methods and therefore propose the need for implementation of classical or hybrid additive–subtractive machining methods incorporated with digital technologies.

Dayam et al. [13] mention the benefits of implementation of sensors and IoT-based automation and communication services because legacy machines have limited or no adaptability to smart manufacturing. Digitalization of the machining processes improves the (operator)–machine–material interactions and helps the operator to achieve guided process control. 

Moreno et al. [14] propose the construction of a digital twin for sheet metal punching machine with a conclusion that there is a necessity of the machining process virtualization of this kind which improves the manufacturing process, while Kurth et al. [15] mention how, by digitalization, higher process reliability can be achieved with implementation of smart machine components which gather and evaluate machine and process data on decentralized spots with certain integrated sensors. 

Akyazi et al. [16] have shown that the Industry 4.0 technologies are invaluable opportunity for the machining tool sector, but this can only be achieved if the highly qualified workforce is reskilling and upskilling the current workforce. Those are technical (IoT, Big Data, AI, etc.), transversal (communication skills, negotiation skills, intrapersonal skills and emphathy, risk management, etc.) and green skills (environmental awareness, energy efficiency, etc.).

Ferreira and Guerra [17] in their research deal with control of dimensional and geometrical requirements of the technical components according to the requirements of Industry 4.0 and emphasize the importance of measuring process connected to large data acquisition to correct the machine parameters and overall product quality.

Singh et al. [18] have given a critical review on ecological, economical, and technological aspects of minimum quantity lubrication towards sustainable machining, which is also one of the characteristics of metal machining in Industry 4.0. They claim that the excessive exposure and usage of fluids leads to an unpleasant environment and uneconomical machining. Water footprints, pollution level, and global warming impact measuring are other challenges Industry 4.0 tends to deal with in this sector, while Canizares and Valero [19] found that the improvements of using IoT technologies in metal machining are very high resulting in the higher efficiency and cost reduction. Maier et al. [20] and Knittel et al. [21] have noticed similar benefits from digitization of the tools. 

Since the deficiency of an optimal and accurate strategy plan has been shown as one of the biggest barriers in Industry 4.0 adoption [22], this readiness factor calculation method will be very useful for the development of local and global manufacturing industry.

This paper consists of an introduction, a literature review where the current state-of-art findings in the field of the readiness factor in Industry 4.0 are examined and through which the scientific gap has been defined needed to formulate the novel readiness factor calculation model, based on the decision support systems, which is described in the following chapters, validated through simulation and proven in a case study according to which the conclusion is given, along with the possibilities of the future research and development in this important topic.

## 2. Literature Review

In order to get an overview of the most relevant and advanced Industry 4.0 readiness factor calculation methods, the Web of Science and Scopus databases were researched by key words “readiness factor”, “maturity”, and “Industry 4.0”. The most important and interesting findings are described in continuation.

The development of advanced readiness assessment models for Industry 4.0 started in 2015, when IMPULS (Foundation of the German Engineering Federation) presented “Industrie 4.0 Readiness” model [23]. It recommended an assessment in six dimensions (employees, strategy and organization, smart factory, smart operations, smart products, and data-driven services) including 18 items to indicate readiness at five levels (outsider, beginner, intermediate, experienced, expert, and top performer). This is an online questionnaire which provides easy and fast self-assessment for the user, but also offers the possibility of creating a benchmark in the industry. At the same time, several similar self-assessment online approaches were presented ([24,25,26]) which evaluated the readiness stage roughly and gave a basic overview of the current practice within the entire company.

Shortly thereafter, Schumacher et al. [27] developed a maturity model which consists of nine dimensions (products, customers, operations, technology, strategy, leadership, governance, culture, and people) assigned to 62 items. The company evaluate their current stage on a scale from 1 (not implemented) to 5 (fully implemented) for each item. Each item was assigned a special weighting factor.

Ganzarain and Errasti [28] presented a “three stage maturity model”, specialized for SMEs. This was a new collaborative diversification methodology that resulted in the opportunity map and company’s business modelling for Industry 4.0 development. The dimensions that were evaluated are energy, electronics, digital business, and advanced metal mechanic in three stages: vision (*envision* 4.0), roadmap (*enable* 4.0), and projects (*enact* 4.0). In the first stage, the current capacity and resources were analyzed, along with the general understanding of Industry 4.0. In the second stage, the requirements were identified as well as the technologies evolved in Industry 4.0. In the final, third stage, the training capacitation was recognized, the risk management was evaluated and the project for the Industry 4.0 implementation developed. The maturity scale was defined in five levels (initial, managed, defined, transform, and detailed business model).

Babić at al [29] have presented a ranking method of enterprises with regard to the industrial maturity level using decision support methods analytic hierarchy process (AHP) and TOPSIS. The expert group of 38 CEOs in Croatia have evaluated the importance of items in three main groups: technique, organization, and personnel. The weighting of the items was done with AHP and later used in the TOPSIS method to calculate the relative closeness index of the company to Industry 4.0. A similar approach was used by Koska et al. [30] in their study.

“SIMMI 4.0 System Integration Maturity Model Industry 4.0” by Leyh et al. [31] enables the classification of IT system landscape of the company and consists of five stages, each of which describes several characteristics of digitization. This approach enables self-assessment of the company with recommendations for activities to be taken in each stage.

Godsell et al. [32] presented “an Industry 4.0 readiness assessment tool” which considers six dimensions (products and services, manufacturing and operations, strategy and organization, supply chain, business model, and legal considerations) with 37 items (sub-dimensions) and rates the companies on four readiness levels (beginner, intermediate, experienc32.ed, and expert). Again, this is an online-based questionnaire and today this is one of the commonly used approaches to study the maturity level of companies in the world.

Felch and Asdecker [33] have developed an Industry 4.0 maturity model for the delivery process in supply chains (DPMM 4.0). The model consists of five stages (basic digitization, cross-department digitization, horizontal and vertical digitization, full digitization, and optimized full digitization) for order processing, warehousing and shipping with the quality criteria consisting of comprehensibility, comprehensiveness, relevance, consistency, systematic structure, detailedness, conceptual reliability, and applicability which are evaluated by the user.

Kaltenbach et al. [34] have measured smart services maturity level in Germany with the conclusion that the maturity level depends mostly on technology management, financial resources, and corporate culture in the company, while Canetta et al. [35] developed a similar digitalization maturity model for manufacturing sector based on dimensions of value drivers, levers of action, processes, and enabling technologies, all of which are based on the maturity calculation by Schumacher et al. [27].

Basl and Doucek [36] presented a “Metamodel for Evaluating Enterprise Readiness in the Context of Industry 4.0”, based on the analysis of previously mentioned methods. They proposed maturity in seven levels, each more detailed than the previous one: society, area of society, branch of area of society, enterprise, area of enterprise, dimension of enterprise area, and sub dimension of enterprise area. The exact calculation method of the maturity index was not revealed in this work. Trotta and Garengo [37] evaluated an average score in dimensions of strategy, technology, production, products, and people through a survey, while Gracel and Lebkowski [38], based on the scientific gaps recognized in previous studies created a “Manufacturing Technology Maturity Model” with eight dimensions (core technologies, people and culture, knowledge management, real-time integration, infrastructure, strategic awareness and alignment, process excellence, and cyber security), but only presented it as a framework for further development.

Oleskow-Szlapka et al. [39] have detected a gap where the maturity calculation method for artificial intelligence (AI) within the industrial system has not yet been developed. Therefore, they proposed a model which combines the maturity levels of both AI and Logistics 4.0. It is based on five dimensions (strategy, organization, data, technology, and operations) and four maturity levels have been defined (AI novice, AI ready, AI proficient, and AI advanced). With this in mind, they proposed a calculation framework that consists of chi-square independence test to verify the relation between the level of maturity and dimensions and consequently suggested using the multi-criteria decision analysis to provide ranking of the dimensions.

Maisiri and Dyk [40] presented a model which combines the Siemens [41] and Impuls (VDMA) [23] approach. The starting point is a questionnaire divided into seven sections, evaluating six main categories (organizational strategy, organizational infrastructure, smart operations, smart products, data-driven services, and employees). Each category was assigned weight based on the previous VDMA research and the organizations were clustered by size. Using ANOVA and *t*-test, the relationships between the categories were examined, while the results were evaluated by basic descriptive statistics methods.

Machado et al., 2019 [42] presented a case study of seven companies in which goal was to define the challenges and enablers towards Industry 4.0. The data was collected with self-check tool previously developed by Lichtblau K. et al. (IMPULS study) [23] which was later evaluation through interviews at an organized workshop. The results have been compared to IMPLUS study. Seven companies of different size participated in this research and the results have shown that there is a need for the prioritizing the steps and actions of the digital transformation, because many are focused on the implementation of digital technology without discussing and evaluating its actual future benefits. Additionally, they forget to focus on other organizational values such as flexibility, quality, or sustainability discussions so the strategic prioritizing of the Industry 4.0 elements would be useful before the actual transformation begins. 

Vrchota and Pech [43] carried out a questionnaire survey on 276 enterprises in Checzh Republic. Based on the results they have calculated the index of Industry 4.0 (VPi4) by explorative factor analysis which was verified by Mann–Whitney test and correlation coefficients. They claim that the VPi4 “enables the enterprises to determine their own level of current state of readiness for Industry 4.0, to better prioritize business development”. They concluded that the more than half of the participating companies feel influenced by Industry 4.0, while 65.7% already have started the implementation process. They have also mentioned human skills as well as the Big Data and Cloud computing technologies and the most important in achieving the higher levels of Industry 4.0.

Pirola et al. [44] developed a “Digital Readiness Level 4.0” model (DRL 4.0), a “specific tool to assess enterprises in relation to manufacturing digitalization”. Apart from the readiness level, the results give suggestions for future development. The model was developed as a multiple-case study approach. In the first phase, the model consists of a questionnaire with 35 questions about five Industry 4.0 dimensions (strategy, people, processes, technology, and integration). Following the interview, the results are evaluated and the average readiness index is calculated.

Lucato et al. [45] recognized that the previously published models did not evaluate the readiness of the companies in the earlier step of maturation process. Hence, they developed a concept based on a standard SAE J4000, which enables consistency and a possibility of comparison between the ideal condition and the current status. Each dimension was evaluated on four possible levels (degrees) of its current implementation status. Dimensions evaluated (Internet of Things, Big Data, Cloud computing, Cyber-physical Systems, Collaborative Robots, Additive Manufacturing, Augmented Reality, and Artificial Intelligence) are the prerequisites of the Industry 4.0 implementation.

Santos and Marthino [46] proposed a maturity model with 41 variables of six dimensions (organizational strategy, structure and culture, workforce, smart factories, smart processes, and smart products and services). The data evaluation was based on the De Bruin et al. [47] maturity model with six iterative stages (scope, design, populate, test, deploy, and maintain) and six maturity levels, based on the judgement of the current implementation level of each variable.

Antony et al. [48] presented a study which has conceptualized the dimensions which are valuable in the evaluation of Industry 4.0 readiness. The research was conducted through an online survey with 37 senior managers participating in the first and 70 in the second phase. Ten dimensions and their criticality for the readiness factor calculation was defined and those are “technology readiness, employee adaptability with Industry 4.0, smart products and services, digitalization of supply chains, extent of the digital transformation of the organization, readiness of Industry 4.0 organization strategy, innovative Industry 4.0 business model, leadership and top management support for Industry 4.0, organizational culture and employee reward and recognition systems”. They concluded that the readiness factor is very beneficial when calculated prior to implementation of Industry 4.0 technologies. 

Wagire et al. [49] proposed an empirically grounded and technology-focused maturity model. It consists of seven dimensions (people and culture, Industry 4.0 awareness, organizational strategy, value chain and processes, smart manufacturing technology, product and service oriented technology, and Industry 4.0 base technology) and 38 maturity items. Fuzzy Analytic Hierarchy Process (FAHP) was used to determine the weights (relative importance) of dimensions and items, and is later multiplied by the score of each item and dimensions.

Kruger and Steyn [50] presented a conceptual model of entrepreneurial competencies needed to utilize Industry 4.0 technologies in order to emphasize the connection between entrepreneurial competencies and novel technologies utilization with Industry 4.0 readiness. The data was collected by conducting interviews with companies and analyzed with machine learning technologies. After transcribing the interviews with the help of AI, the text was inserted into ATLAS.ti and Voyant Tools software to identify the relationships between the categories which were later rearranged into a hierarchical form.

Sriram and Vinodh [51] have analysed the already available readiness models for SMEs and by using multi-criteria decision-making method COPRAS they ranked the dimensions by priority.

Caiado et al. [52], with the development of the fuzzy rule-based Industry 4.0 maturity model, tried to eliminate human subjectivity in the evaluation process. They implemented fuzzy logic and Monte Carlo simulation into Industry 4.0 self-assessment tool, which they later validated in a case study. With an in-depth literature review they identified the dimensions (criteria for the evaluation), and afterwards designed the model using interviews and focus groups.

So far, this is the most complex Industry 4.0 readiness assessment model available in the literature.

As the most recent and complex study, its authors mentioned the limitations of the model related to the consideration of group decision-making with multi-criteria decision support methods to solve possible different views of the decision-makers involved in the process.

### Scientific Gap

From a detailed literature review, it is clear that most of the readiness factor calculation models provide a result which describes the general position of the company as a single system, compared to the ideal Industry 4.0 characteristics. Very few models are specialized for a single phase of the manufacturing process or a single manufacturing department. Process planning, as one of the most important stages in product manufacturing has not yet been a topic of a specialized readiness factor study and model definition. Furthermore, the readiness factor calculation demands a detailed evaluation of a various number of specific criteria, where each has a certain importance (weight). That is why as part of this research the model for readiness factor calculation oriented to process planning based on the decision support method will be developed as an innovative method for an optimal future transitional strategic plan definition and as a valuable and accurate managerial tool which focuses on small and medium enterprises.

## 3. Methodology

**Figure 1:**
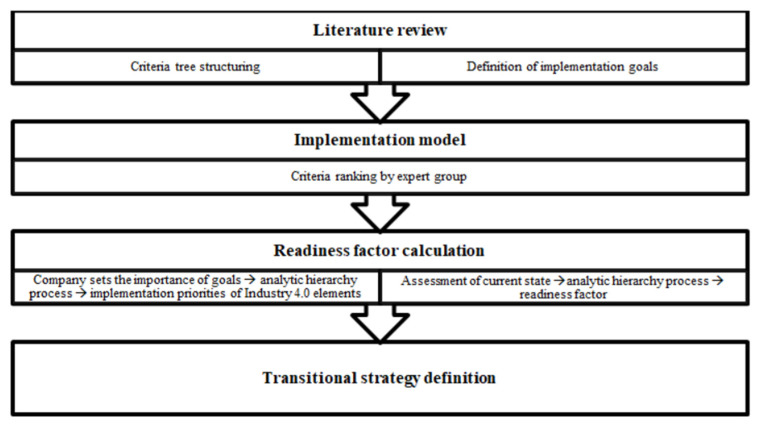
Framework of the innovative, decision support-based readiness factor calculation model.

With the available findings from the literature in mind and the recognized research gap, the model for calculation of the readiness factor, specialised in process planning, has been defined and based on multi-criteria decision support methods. The framework of the readiness factor calculation process method is shown in Figure 1.

As shown in Figure 1, through literature review the criteria tree is structured and those are elements of Industry 4.0 most commonly mentioned, as well as the elements of ideal model of process planning using the Industry 4.0 concept [34]. Similarly, the goals for the Industry 4.0 implementation are defined, representing the target which the company expects to achieve with digitization. The criteria are assigned (Industry 4.0 elements) weights by an expert group from the field, consisting of 30 experts, 15 from academia, and 15 from the industry. They evaluate the priority and importance of its implementation according to each of the goals set. This is the implementation model which shows the ranks of importance of Industry 4.0 elements by priority of its implementation, specialized for process planning. The model is structured according to principles of analytic hierarchy process (AHP method) in which, in the first phase, Industry 4.0 elements are set as alternatives and goals as criteria. The data input in this phase are goal preferences of a single company and the output is the rank of the elements according to the need for optimal implementation. To calculate the readiness factor and generate optimal transitional strategy, the ranks of Industry 4.0 elements from the previous phase are modelled in the analytic hierarchy process as criteria with two possible alternatives—the current state and the ideal state of the company. Evaluation of these alternatives is set by results from the questionnaire filled in by company representatives in which each question is linked to each Industry 4.0 element. The results are normalised, and the readiness factor is calculated in comparison to the ideal state. The detailed mathematical procedure will be described in the following chapters.

### 3.1. Definition of Process Planning Oriented Industry 4.0 Elements and Goals

The elements of Industry 4.0 that are process planning oriented as well as the implementation goals have been defined by reliable literature sources according to the dynamics of their appearance. The frequency of the most common goals and elements is shown in Table 1 and Table 2.

In Table 1, the most common goals from the literature are shown with the references to their appearance, while in Table 2 the most common elements of Industry 4.0 presented are those used as a criterion in readiness factor calculation methods described in the previous chapter.

Therefore, according to the frequency of appearance in the literature and the authors’ contribution to the adaptiveness for the process planning, the following goals are defined:

1. Increase of productivity
2. Increase of product quality
3. Readiness for financial investment
4. Complexity of execution and application
5. Expected return of investment time.

The goals represent the target which the company aims to achieve once the new digital concept is implemented. Elements of Industry 4.0 represent the dimensions for the evaluation with certain weights. They were also adapted for the needs of process planning and divided into three groups—“smart process planning”, “infrastructure”, and “organization and human resources”. This is how the evaluation/readiness factor calculation can be oriented to a single specific field (in this case, process planning), but it also considers the elements which are indirectly connected to process planning but very important and needed for its digital transformation. The defined criteria are shown in Table 3.

The first group “smart process planning” contains the most common elements directly connected to process planning in a digital environment, such as automatic recognition of geometrical features or automatic definition of a manufacturing plan. In the second group, “infrastructure” there are criteria needed as support for normal functioning of the criteria from group 1, such as internet infrastructure, or real-time data collection and archiving in databases. The third group “organization and human resources” covers human-oriented elements (dimensions) such as the education level of the worker or their innovation level.

### 3.2. Implementation Priorities (Criteria Weighting) and Model

In general, the readiness factor gives an overview of the current development level of the company and compares it with the ideal development level according to the Industry 4.0 concept principles.

The novelty model for the readiness factor calculation is based on the decision support systems and oriented towards process planning. That is the reason why, after the definition of the criteria tree, weights must be assigned to each criterion. Weighting was provided by an expert group which consists of 30 experienced professionals in the field of process planning; 15 of which are from academia and 15 from the industry. They have rated the elements by priority (on a scale of 1 to 9, where 1 is the lowest and 9 the highest given priority) for each goal. The ranks therefore provide the priority list of Industry 4.0 elements by order of optimal implementation if a certain goal is to be achieved, as part of an optimal development strategy. Since most of the companies will attribute different importance to certain goals, the model is formed according to principles of analytic hierarchy process decision support method. To obtain an optimal priority list for the implementation of certain Industry 4.0 elements, the goals are the criteria in the AHP tree, while the elements of Industry 4.0 are the alternatives.

**Figure 2:**
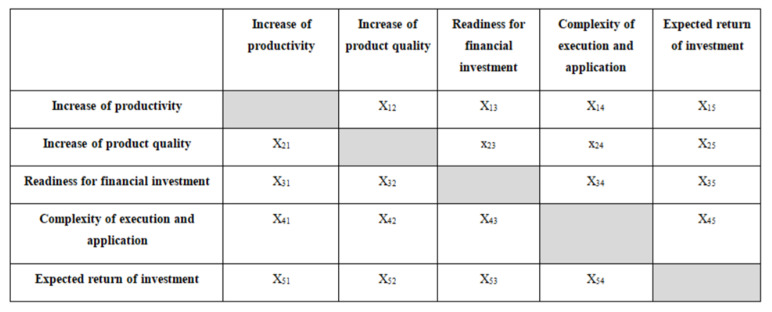
Priority matrix of the goals.

In Figure 2, the priority matrix of the company’s preferences is shown. It requires the input data provided by the company representative who evaluates the importance of each goal for the company’s future state. According to the AHP method, the importance is set in pairwise comparison on a scale from 1 to 9, where 1 is the lowest and 9 the highest importance, which can be illustrated with a priority matrix and later used for further steps of calculation.

The weights of the alternatives (Industry 4.0 elements) are defined by the expert group in the previous phase and calculated through the ranking method common in the Friedman test, while the ranks are calculated into the weights through the normalized vector method.

**Figure 3:**
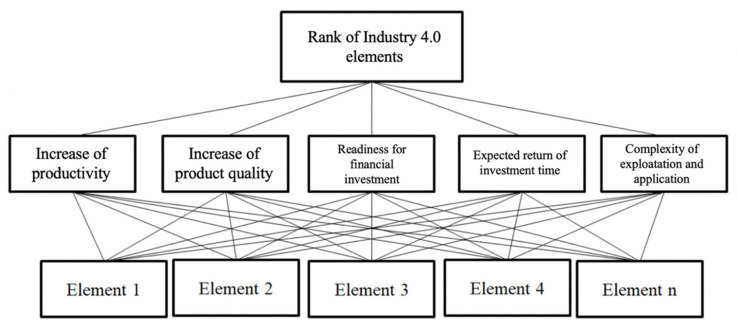
Structure of the implementation model for Industry 4.0 elements oriented to process planning.

The problem structuring according to the principles of the AHP method in which goals are set as criteria and elements as alternatives is shown in Figure 3. According to the input data of importance of goals with weight of each element defined by an expert group, the results are defined as a rank list in which the first suggested alternative (element) as optimal result is taken as a suggestion to be implemented first, followed by others by rank.

The mathematical procedure where the input data are the goal and element weights with output data as ranks of elements is shown in (1).

(1) $$\left[\begin{array}{ccccc} a_{11} & b_{12} & c_{13} & d_{14} & e_{15}\\ a_{21} & b_{22} & c_{23} & d_{24} & e_{25}\\ \ldots & \ldots & \ldots & \ldots & \ldots \\ a_{i1} & b_{i2} & c_{i3} & d_{i4} & e_{i5} \end{array}\right]*\left[\begin{array}{c} f_{11}\\ f_{21}\\ f_{31}\\ f_{41}\\ f_{51} \end{array}\right]=\left[\begin{array}{c} g_{11}\\ g_{21}\\ \ldots \\ g_{i1} \end{array}\right]$$

where:

*a*_ij_—weights of return of investment time

*b*_ij_—weights of product quality increase

*c*_ij_—weights of product productivity increase

*d*_ij_—weights of readiness for financial investment

*e*_ij_—weights of implementation and exploitation

*f*_ij_—values of company priority vector calculated by the AHP method

*g*_ij_—ranks

*i*—number of Industry 4.0 elements

j = 5—number of criteria

The weights (priorities, ranks) of the Industry 4.0 elements from the previous step are defined as the priority vector of the company, which multiplied by the vector of the evaluation of the current environment give the final readiness factor for each of the three groups (2).

(2) $$\left[\begin{array}{cccc} x_{11} & x_{12} & \ldots & x_{1i} \end{array}\right]*\left[\begin{array}{c} y_{11}\\ y_{21}\\ \ldots \\ y_{i1} \end{array}\right]=F_{j}$$

where:

*x*—evaluation of the current working environment through each Industry 4.0 element

*y*—rank of each criterion (Industry 4.0 element)

*i*—number of Industry 4.0 elements

*j*—group of Industry 4.0 elements

Finally, the overall readiness factor is calculated by adding weights to each group and multiplication of each readiness factor (3).

(3) $$F=\sum_{j=1}^{n}F_{j}*z_{j}$$

where:

*F*—overall readiness factor of the company for Industry 4.0. It can take on value from 0 to 1, where the ideal company always has the value 1

*z*—weight of each element’s group.

**Figure 4:**
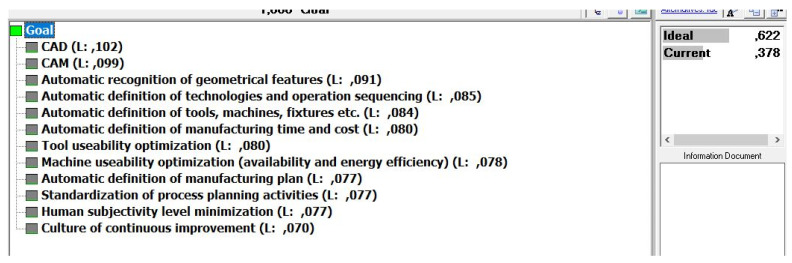
Readiness calculation model structuring in Expert Choice software.

This means that the model for the readiness factor calculation by the AHP principles is structured so that the elements of Industry 4.0 with rank weights from the previous step are now the criteria with only two possible alternatives—the ideal state and the current state (Figure 4). This was provided by Expert Choice software and the normalized results from the questionnaire are filled in as weights of the alternatives directly. This is how human subjectivity is minimized and the criteria and alternative evaluation process quantified.

The performance of the model was verified by providing the what-if analysis and simulation in which the predicted input has generated the predicted output data and the minor changes up to 5% have not caused the difference in the element ranks which validates the robustness of the model.

## 4. Results

The ranking of the elements of Industry 4.0 is done according to the following principles:

- implementation of elements that enable higher productivity, which means that the elements which increase productivity have a higher weight
- implementation of elements that enable higher product quality, which means that the elements which affect the increase of product quality have a higher weight
- implementation of elements where the company is more willing to invest financially, which means that the elements in which the companies are more willing to invest have a higher weight
- implementation of elements with less complexity of execution and application, which means that the elements which are simpler for execution and application have a higher weight
- implementation of elements with a shorter return of investment time, which means that the elements with a shorter ROI time have a higher weight

The ranks of the elements of groups of criteria by certain goal is shown in Table 4, Table 5 and Table 6.

### 4.1. Readiness Factor Calculation

**Figure 5:**
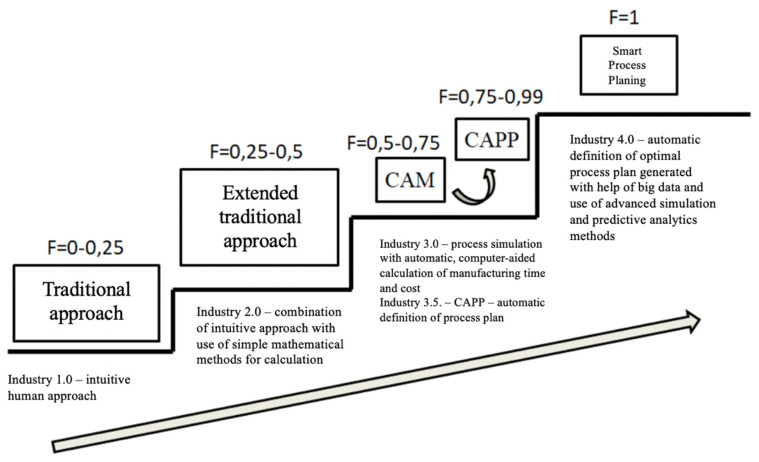
Readiness factor and digitization level of process planning.

In the next step, to obtain a quantified result for the readiness factor and to be able to define the strategy plan accurately, the company representative is asked to fill in the questionnaire in which every element of Industry 4.0 from the three groups is evaluated. Each question has five possible answers defined by the level of their current development, as shown in Figure 5. The results are therefore quantified and normalized, calculated on a scale from 0 to 1, where 1 is the ideal level of development and 0 means that there is no sign of presence of the element in the current working environment.

The readiness factor, as shown in Figure 5, is defined in the following levels:

0–0.25: Traditional approach. Their work is based by Industry 1.0 principles, which includes a manual definition of the manufacturing plan by an intuitive approach of a process planner.

0.25–0.5: Extended traditional approach. Manufacturing principles are based on Industry 4.0 characteristics, which is the intuitive manual approach with the additional use of simple mathematical methods in the process planning for the definition of time and cost.

0.5–0.75: CAM. Related to the third Industrial revolution, the use of computer is essential, especially when defining the manufacturing plan, by using tools like CAD or CAM for the computer-oriented automatic time and cost calculation and the definition of certain manufacturing plan data from the computer database.

0.75–0.99: CAPP. Characteristics of Industry 3.5, which is the last step before a complete digitization. This includes the use of advanced CAPP systems.

1: Smart process planning, based on the Industry 4.0 principles. Digital concept is fully implemented.

### 4.2. Case Study

The functionality, useability, and reliability of the process planning oriented readiness factor calculation model is proven on a case study in a Croatian metal machining company which produces robotics components with a strategic tendency towards automation and digitization of the manufacturing processes.

The company is familiar with the Industry 4.0 concept and considers its implementation to be one of the future highly important goals. The company representative was the CEO who has long-term experience in the process planning field.

**Figure 6:**
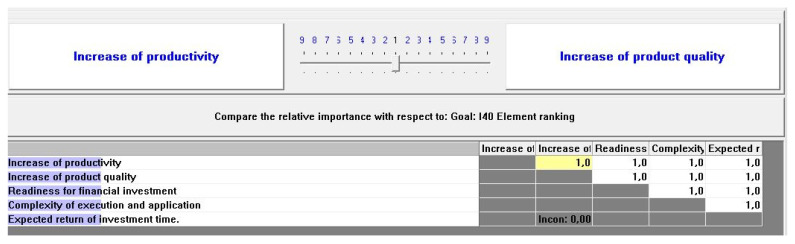
Goals importance definition—case study.

**Figure 7:**
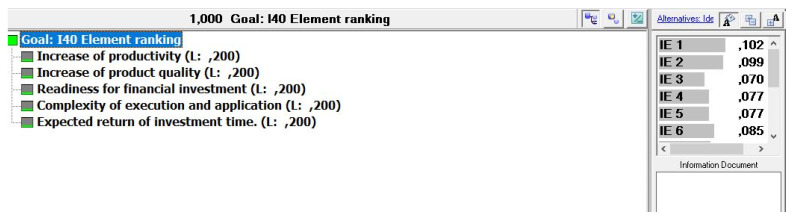
Single element (criterion) evaluation—model.

**Figure 8:**
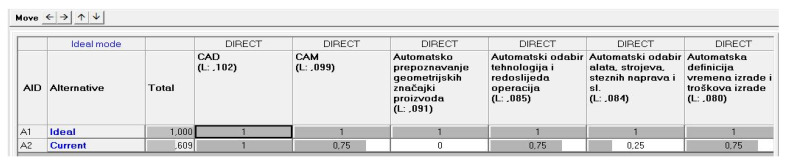
Single element (criterion) evaluation—data input.

**Figure 9:**
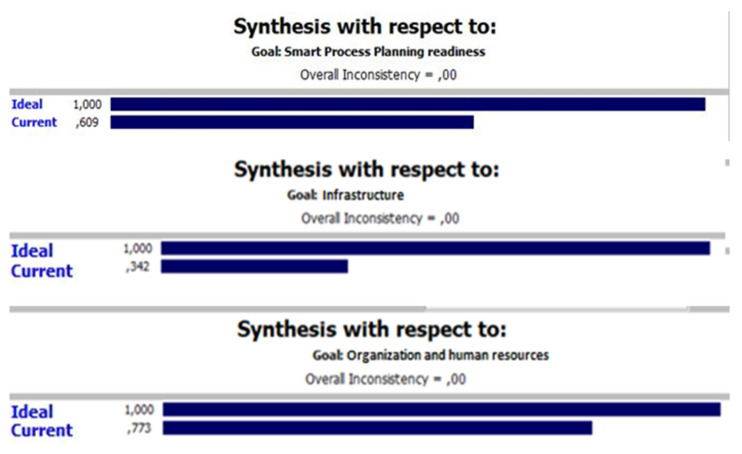
Readiness factor results by group.

The goal priorities they set was for each to have an equal weight (Figure 6). Therefore, according to the procedures described in the previous chapter, the priorities for the implementation of elements are generated. The next step involved the evaluation of each criterion, its comparison to the ideal target state, according to which the readiness factor was finally calculated (Figure 7 and Figure 8). For the “smart process planning” group of the elements the readiness factor was 0.609, for the “infrastructure” 0.342 and “organization and human resources” 0.773 (Figure 9). Each of the three groups also has weighting importance weights (Table 7), which were provided by the results obtained in the previous research by the expert group [67] and which were defined again by the normalized vector method (Table 5). Therefore, to get a final readiness factor, each of the separate factors for the group was multiplied by its weight, described in (3). The overall readiness factor of this company is 0.5927, which shows that this company belongs to the group of development level similar to the Industry 3.0 characteristics. Since the factor range for Industry 3.0 in this case is from 0.5 to 0.75, it can be concluded that the actual state is closer to Industry 2.0 than 3.0.

### 4.3. Discussion—Implementation Strategy

When defining the implementation strategy, it is necessary to consider the results of the evaluation of the current situation. Elements that received a grade of 1 (5) and are thus equated with the ideal company, should not be implemented or improved, when there is a basic form of these in the company, namely “CAD” in the group “smart process planning”, and “employee motivation”, “innovation of workers”, “acceptance of the principles of lifelong learning”, and “readiness of workers for change” in the group “organization and human resources”. In the group “infrastructure”, no element received the highest grade. Therefore, the elements according to the priorities of introduction are shown in Table 8.

The highest priority is given to the criteria from the group “organization and human resources”, so it is recommended to first adopt the principle of continuous improvement, then work on connectivity and vertical integration as well as decentralization of the company. Next in importance are the elements from the group “infrastructure”, among which the priority is the development of advanced databases. There follows the need for modularity and flexibility of the software system, using the predictive analytics methods, state-of-the-art Internet infrastructure and Cloud computing where the system, both software and hardware, should be maintained by predictive maintenance methods which should be enabled in optimal form and assigned greater significance. Furthermore, it is necessary to improve the computer infrastructure, raise the level of data and system security, implement the ERP system, enable better connectivity so that, finally, a large amount of data can be optimally collected, processed, and stored in real time.

The following are the elements from the “smart process planning” group, the primary of which is the improvement of the CAM system and the adoption of the habit of monitoring and introducing new technological trends, with continuous monitoring and optimization of the system. Then it is necessary to automate the definition of time and cost of production and standardize design activities in order to approach the optimization of tool usage and energy efficiency of machines, minimize the impact of subjectivity of technologists and achieve automatic definition of the entire design plan. The smart process planning system is complemented by the implementation of procedures for automatic selection of technologies, machines, clamping devices, and geometric recognition of the product features based on 3D models, as one of the most complex elements of this group.

In such manner, with the expressed priorities of the company’s goals, an optimal transition from the current situation to the ideal can be achieved—i.e., the concept of Industry 4.0 can be fully adopted when designing technological processes.

Felch et al. [33], Gracel and Lebkowski [38], and Antony et al. [48] have recognized the difference in the implementation of Industry 4.0 elements in different manufacturing sectors. This is, once again, confirmed in this research in where the model for the readiness factor calculation is oriented to process planning and the priorities for implementation of Industry 4.0 elements are defined by certain goals company aims to achieve in the future, specially in this field.

Kaltenbach et al. [34] mentioned that one of the most important findings from their case study is the aim that the operative workers will be substituted by technologies. Digitization in general is aimed towards of the human operative work elimination where a worker gets another role of a controller and developer and in this research process planning activities, which include not only the operative work, have been digitized, so the process plan can be generated automatically. They have also noticed that there was no clear understanding about the topics and technologies of Industry 4.0 in the manufacturing, as one of the biggest barriers in implementation. Automated work, in their research has a highest weight, followed by simultaneous engineering and future plans. Similarly to presented research, in which the automation of process planning has a highest priority, but also the personalized strategy definition which is of the presented model.

The importance of optimal strategy definition, as one of the most important steps before implementation of Industry 4.0 elements was mentioned also by Caneta et al. They have also emphasized a special worker’s skills because of the modification of the activities. That is why in this research the “organization and human resources” group has been specially developed and given a highest weight to, in which for each goal the highest priority was given to criteria “high innovativeness of the workers”, “life-long learning principles”, “workers’ readiness for change”, and “high motivation of every worker”. The understanding of the path towards Industry 4.0 and the concept itself was also one of the key findings of Pirola et al. [44] but also Santos and Martinho [46] and Wagire et al. [49]. Similarily, Jones et al. mention the importance of the new mindset in the company, with focus on the innovativeneess, collaboration, and experiments, and mention mindset as one of the most important barriers in the implementation process. 

Machado et al. [42] also mention the importance of knowledge so that the companies should increase efforts on training and identifying internal competencies, but also the importance of implementation of data-driven processes. In presented research the “infrastructure” group of criteria, which is largely based on components that enable data-driven processes has given a second highest priority, while inside the group the criteria related to data manipulation and analysis were recognized as most important in every goal.

On the other hand, Maisiri et al.’s [40] research has given the highest importance to transformation of operations, followed by infrastructure while the organizational strategy and employees were not a top priority. 

Basl and Doucek [36] mention the highest importance of the cyber security dimension which enables the normal functioning of the other elements, while this model finds this criterion the most important when achieving a goal “expected return of investment time”. 

Antony et al. [48] claim that the technology readiness of the organization depends on how well an organization is ready to implement certain elements but depending on the objectives of the organization, as it was shown in this research by five possible different goal definitions to which the variable importance can be given. However, they mention that social components are as important as technology.

### 4.4. Limitations

Most of the usual limitations of the AHP method are avoided in this case because of the cautious model structuring with the goal to eliminate the most common limitations. The first one is the possible appearance of inconsistency during the pairwise comparison of criteria and alternatives. The possibility of inconsistency increases with a larger amount of criteria or alternatives in the decision tree. In the presented model this was avoided by definition of the weights of the criteria (Industry 4.0 elements) with statistical ranking used in the Friedman test, while the ponders implemented in the model (decision tree) have been calculated by normalized vector method. This kind of limitation remains in the phase of goal setting when the company needs to decide about the importance of each goal in the model’s decision tree defined as criteria. To avoid this, since the readiness factor is being generated independently as a personalized calculation of a single company, the supervisor from the evaluating team should remain in contact with the company and provide guidance in this step so it does not influence the accuracy of the following steps of the readiness factor calculation and strategy development. According to Saaty, the acceptable inconsistency rate can be 0.1 [68]. 

Another limitation of the AHP method is the high influence of human subjectivity in the decision-making process. In the presented model, this was avoided by forming the expert group which enabled the increase of the accuracy of the core data needed for the model (Industry 4.0 elements) which are in the first step of the readiness factor calculation defined as alternatives in the decision tree. In the second step, there is an influence of human subjectivity when the company needs to evaluate the current state of their work environment which is later compared to the ideal. In the model this was minimized by the detailed criteria definition in which the human subjectivity is minimized because of the detailed description given in the questionnaire which makes their personal judgment accurate. The accuracy in this step can be increased by adding multiple representatives of the company who would participate in the readiness factor calculation and strategy definition process.

### 4.5. Scientific Contribution

The results have shown that with use of decision support systems in readiness factor calculation minimization of human subjectivity in the readiness factor calculation can be achieved. The qualitative criteria evaluation criteria have been quantified which increases the accuracy of the results. The previously presented models in the literature as an output give a number which defines the current position of the company compared to the ideal state, while this model goes a step further and gives an important strategic plan which is the priority list of the Industry 4.0 for the implementation with the optimal benefits in the future, according to the goals a company has set. Additionally, this model has given a detailed structure of the elements that need to be implemented for the digital transformation of the process planning, which is a novelty in the literature. The readiness factor calculation method oriented to process planning has not yet been presented in the literature, so this model is a useful scientific contribution to the theory and practice.

### 4.6. Managerial Implications

Industry 4.0 readiness factor calculation model oriented to process planning presented in this paper enables the simple definition of optimal transitional strategy definition. Most of the previously mentioned models from the literature are structured with goal to examine the current state of the entire company by most important Industry 4.0 elements. This model is oriented to process planning, based on the ideal model of process planning in the digital environment. The readiness factor is a quantitative value by which the company can get an overview about its position within the other competitors in the same or similar industry, while the transitional strategy gives a very useful and optimal information about the implementation priorities of Industry 4.0 elements specifically created by the goals and benefits which a company aims to achieve in the future by implementing Industry 4.0.

## 5. Conclusions

The novel and innovative readiness factor calculation method based on decision support systems, in this case the analytic hierarchy process, is a very effective and accurate tool in the definition of a strategic plan for the digital transition towards the implementation of Industry 4.0. With the use of knowledge provided being evaluated through statistical methods by an experienced expert group, the influence of human subjectivity is minimized and the evaluation of qualitative criteria in the readiness factor calculation method quantified, which answers RQ3. Human subjectivity can not be eliminated, but it can be minimized, regarding even the limitations of the AHP method. This model is process planning-oriented, meaning that it provides a detailed overview of the current state in this very important phase of the product lifecycle, which greatly benefits the manufacturing industry community because a detailed and specialized model for this phase has not been presented yet. The readiness factor describes the distance between the current state and the wanted, ideal stage of digital manufacturing, built by the Industry 4.0 concept. Based on the research findings from the relevant literature and expert group experience, the detailed criteria tree has been defined in three groups: “smart process planning”, which is based on specific tools and methods used in process planning; “infrastructure”, which is based on the infrastructure elements in the company which enable the process planning activities and “organization and human resources”, which is based on the organizational and human resources features of the company, and is also very relatable to the process planning and the successful implementation of the new and innovative business model. The readiness factor was calculated with the help of Expert Choice software, the model was proven by what-if and sensitivity analysis and its usefulness was proven as effective on a case study on a metal machining company, which provides an answer to RQ2. The result is an optimal strategic plan, according to the goals which the company aims to achieve with Industry 4.0, as the priority list for the implementation of the Industry 4.0 elements for each criteria group—which so far overcomes the most common and complex implementation barrier yet—the lack of a strategic plan, which, finally, answers RQ1.

For the future research, this kind of model can be extended or developed for various industrial phases, such as manufacturing, logistics or product design. This is a step towards the definition of a complete and detailed criteria tree which would enable an accurate, quantitative, and optimal readiness factor calculation and strategy definition for the entire manufacturing company.

## Figures and Tables

**Table 1:** Most common goals for the Industry 4.0 implementation in literature.

Goal	Source
Strategy	[32]; [53]; [11]; [36]; [12]; [37]; [54]; [55]; [56]; [34]; [57]
Investment and business model	[40]; [42]; [16]; [44]; [12]
Increase of product quality	[45]; [36]; [37]; [38]; [35]
Reducing the costs	[42]; [16]; [40]; [44]
Decrease of manufacturing time	[16]; [43]
Increase of productivity	[43]

**Table 2:** Most common elements of Industry 4.0 in literature.

Element (Dimension)	Source
Manufacturing process automation and digitization	[58]; [42]; [16]; [59]; [44]; [43]; [8]; [45]; [11]; [20]; [29]; [60]; [37]; [61]; [22]; [19]; [39]
Smart factory	[40]; [62]; [16]; [40]; [44]; [43]; [8]; [45]; [11]; [14]; [42]; [42]; [39]; [52]
Big Data analytics	[40]; [42]; [16]; [40]; [63]; [8]; [45]; [47]; [22]; [39]
Connection with outer value chain members	[40]; [42]; [40]; [44]; [20]; [12]; [42]; [47]; [37]; [42]; [38]; [18]
Organization	[40]; [42]; [27]; [20]; [13]; [37]; [42]; [39]
IT connection/Internet infrastructure	[40]; [42]; [16]; [40]; [44]; [15]; [17]
Smart products	[40]; [42]; [11]; [20]; [14]; [21]; [42]; [37]; [15]; [18]; [39]
Technologies	[36]; [14]; [21]; [42]; [37]; [15]; [42]; [22]; [38]
Cyber security	[40]; [42]; [16]; [40]; [8]; [45]; [37]; [22]
Cloud computing	[40]; [42]; [16]; [40]; [64]; [43]; [45]; [12]
Education of workers and life-long learning principles	[40]; [42]; [16]; [40]; [43]; [13]; [37]; [19]
Real-time data exchange	[40]; [42]; [11]; [13]; [39]
Real-time data storage	[40]; [42]; [16]; [40]; [45]; [21]; [15]; [17]
Simulation/digital twin/augumented reality	[40]; [42]; [16]; [40]; [44]; [43]; [24]; [37]; [18]
Artificial intelligence/cyber-physical systems	[16]; [40]; [44]; [43]; [8]; [65]; [42]; [47]
Predictive analytics	[40]; [42]; [16]; [44]; [43]; [8]; [12]
Horizontal integration	[42]; [16]; [44]; [8]; [15]; [47]; [33]
Logistics 4.0	[16]; [45]; [30]; [13]; [38]
Digital culture	[42]; [44]; [20]; [13]; [38]; [19]
Vertical integration	[40]; [43]; [15]; [47]; [17]
Advanced technology use, additive manufacturing	[40]; [44]; [43]; [8]; [45]
Smart scheduling and planning	[16]; [8]; [45]; [38]; [39]
Motivation	[42]; [12]; [19]
Innovation	[42]; [40]; [14]; [37]
Decision support	[16]; [44]; [21]; [47]
System self-optimization	[16]; [17]
Energy efficiency	[44]; [43]; [45]
System flexibility	[16]; [43]; [37]
ERP systems	[66]; [22]
PLM	[28]
Predictive maintenance	[43]
Decentralization	[45]; [21]
Renewable energy sources	[45]
Mass customization	[14]
Continuous improvement	[13]

**Table 3:** Criteria for process planning-oriented readiness factor calculation.

Smart Process Planning	Infrastructure	Organization and Human Resources
CAD CAM Automatic recognition of geometrical features Automatic definition of technologies and operation sequencing Automatic definition of tools, machines, fixtures etc. Automatic definition of manufacturing time and cost Tool useability optimization Machine useability optimization (availability and energy efficiency) Automatic definition of manufacturing plan Standardization of process planning activities Human subjectivity level minimization Culture of continuous improvement	Real-time data collection in databases Archiving all data from the manufacturing plan in database Use of data from database when defining new manufacturing plan Use of predictive analytics methods Connection with outer databases Big Data manipulation Excellent computer infrastructure Flexible and modular hardware Flexible and modular software Excellent Internet infrastructure omni available Cloud computing ERP systems High level of data and connection security Predictive maintenance of hardware and software	Excellent connectivity with every part of value chain Special and highly effective communication channels (social networks) Decentralization High motivation of workers Readiness for change High innovation level Life-long learning principle Continuous improvement culture acceptance Horizontal and vertical integration

**Table 4:** Ranks of the elements from the “smart process planning” group.

	Increase of Productivity		Increase of Product Quality		Readiness of Financial Investment		Complexity of Execution and Application		Expected Return of Investment Time	
Industry 4.0 element	Rank	Weight	Rank	Weight	Rank	Weight	Rank	Weight	Rank	Weight
CAD	8.4667	0.1085	8.0167	0.1028	8.5333	0.1094	7.7500	0.0994	7.1000	0.091
CAM	8.1667	0.1047	8.3333	0.1068	7.7667	0.0996	7.9333	0.1017	6.4333	0.0825
Automatic recognition of geometrical features of product	5.9500	0.0763	6.3667	0.0816	4.9333	0.0632	4.2667	0.0547	5.7333	0.0735
Automatic definition of manufacturing technology and operation sequencing	6.8333	0.0876	6.2000	0.0795	5.3667	0.0688	6.0500	0.0776	5.5167	0.0707
Automatic definition of tools, machine tools, fixture, etc.	6.5833	0.0844	5.5667	0.0714	6.6167	0.0848	5.4833	0.0703	5.7167	0.0733
Automatic definition of manufacturing time and cost	7.2833	0.0934	6.0500	0.0776	6.8833	0.0882	6.5667	0.0842	6.2667	0.0803
Tool useability optimization	5.9000	0.0756	6.6000	0.0846	4.7333	0.0607	6.7333	0.0863	7.1667	0.0919
Machine tools useability optimization (availability and energy efficiency)	6.3000	0.0808	4.8167	0.0618	7.0333	0.0902	7.3000	0.0936	6.1667	0.0791
Automatic definition of process plan	4.7833	0.0613	5.8833	0.0754	6.8667	0.088	6.1000	0.0782	6.3500	0.0814
Process planning activities standardization	5.8500	0.0750	6.6333	0.085	6.8167	0.0874	6.7667	0.0868	6.7833	0.087
Human subjectivity minimization	4.4167	0.0566	6.4167	0.0823	4.9500	0.0635	6.8500	0.0878	7.6500	0.0981
Continuous monitoring, optimization of the system and improvement	7.4667	0.0957	7.1167	0.0912	7.5000	0.0962	6.2000	0.0795	7.1167	0.0912
Σ	78	1	78	1	78	1	78	1	78	1

**Table 5:** Ranks of the elements from the “infrastructure” group.

	Increase of Productivity		Increase of Product Quality		Readiness for Financial Investment		Complexity of Execution and Application		Expected Return of Investment Time	
Industry 4.0 element	Rank	Weight	Rank	Weight	Rank	Weight	Rank	Weight	Rank	Weight
Real-time data collection in databases	9.0167	0.0859	8.1167	0.0773	8.4333	0.0803	7.9667	0.0759	7.8000	0.0743
Archiving of all data from process plans to bases	8.1833	0.0779	8.3333	0.0794	8.1500	0.0776	8.6833	0.0827	8.1667	0.0778
Use of data from the base in new process plans	6.8667	0.0654	7.7667	0.0740	8.2667	0.0787	7.6667	0.0730	7.4000	0.0705
Use of predictive analytics methods	9.3500	0.0890	8.6167	0.0821	6.9167	0.0659	7.0500	0.0671	7.6833	0.0732
Connection with external databases	5.9833	0.0570	6.9667	0.0663	6.9000	0.0657	7.5167	0.0716	6.3333	0.0603
Big Data manipulation	6.4500	0.0614	9.2167	0.0878	6.1167	0.0583	5.9000	0.0562	7.6500	0.0729
Excellent computer infrastructure	8.0167	0.0763	6.8167	0.0649	7.7167	0.0735	6.8500	0.0652	6.2500	0.0595
Flexible and modular hardware solutions	7.6333	0.0727	8.1833	0.0779	7.4833	0.0713	7.0167	0.0668	7.3667	0.0702
Flexible and modular software solutions	9.0833	0.0865	7.9167	0.0754	8.2667	0.0787	7.7833	0.0741	7.2500	0.0690
Excellent Internet infrastructure omni available	8.4500	0.0805	6.9500	0.0662	8.2000	0.0781	8.4000	0.0800	7.4333	0.0708
Cloud computing	6.9833	0.0665	6.4833	0.0617	8.0333	0.0765	7.8333	0.0746	7.3833	0.0703
ERP systems	6.4167	0.0611	6.4167	0.0611	6.1167	0.0583	7.3167	0.0697	8.3500	0.0795
High data and network security	6.7667	0.0644	6.1833	0.0589	6.3833	0.0608	7.4833	0.0713	8.3500	0.0795
Predictive maintenance of hardware and software	5.8000	0.0552	7.0333	0.0670	8.0167	0.0763	7.5333	0.0717	7.5833	0.0722
Σ	105	1	105	1	105	1	105	1	105	1

**Table 6:** Ranks of the elements from the “organization and human resources” group.

	Increase of Productivity		Increase of Product quality		Readiness for Financial Investment		Complexity of Execution and Application		Expected Return of Investment Time	
Industry 4.0 element	Rank	Weight	Rank	Weight	Rank	Weight	Rank	Weight	Rank	Weight
Excellent connection with every part of value chain	4.7000	0.1044	4.4833	0.0996	5.1000	0.1133	4.5667	0.1015	4.2167	0.0937
Special and highly efficient communication channels (social networks)	2.9833	0.0663	3.3833	0.0752	4.1667	0.0926	6.6167	0.1470	5.5833	0.1241
Decentralization	2.9833	0.0663	2.7500	0.0611	3.6167	0.0804	5.1167	0.1137	4.8167	0.1070
High motivation of every worker	5.9000	0.1311	6.3833	0.1419	5.2000	0.1156	5.3000	0.1178	5.3667	0.1193
Workers’ readiness for change	5.7500	0.1278	5.8500	0.1300	6.3667	0.1415	4.0167	0.0893	5.2333	0.1163
High innovativeness of workers	6.0833	0.1352	5.7833	0.1285	5.3167	0.1181	4.1500	0.0922	5.2500	0.1167
Life-long learning principle	6.1500	0.1367	5.6833	0.1263	5.6167	0.1248	5.0833	0.1130	5.3833	0.1196
Continuous improvement principle (lean, kaizen)	5.6000	0.1244	6.3667	0.1415	5.3500	0.1189	5.2500	0.1167	4.9667	0.1104
Horizontal and vertical integration	4.8500	0.1078	4.3167	0.0959	4.2667	0.0948	4.9000	0.1089	4.1833	0.0930
Σ	45	1	45	1	45	1	45	1	45	1

**Table 7:** Weighting of goals.

	Average Rank	Sum of Ranks	Mean	Std. Dev.	Weight
PPTP	1.7333	52.000	1.8000	0.7144	0.2889
Infrastructure	1.8500	55.500	1.9000	0.6618	0.3083
Organization and Human Resources	2.4167	72.500	2.4667	0.8604	0.4028
Σ	6.0000				1

**Table 8:** Industry 4.0 elements strategic implementation priorities for process planning—case study.

Rank	Organization and Human Resources	Rank	Infrastructure	Rank	Smart Process Planning
1	Continuous improvement culture acceptance	1	Archiving all data from the manufacturing plan in database	1	CAM
2	Excellent connectivity with every part of value chain	2	Real-time data collection in databases	2	Culture of continuous improvement
3	Special and highly effective communication channels (social networks)	3	Flexible and modular software	3	Automatic definition of manufacturing time and cost
4	Horizontal and vertical integration	4	Use of predictive analytics methods	4	Standardization of process planning activities
5	Decentralization	5	Excellent Internet infrastructure omni available	5	Tool useability optimization
		6	Use of data from database when defining new manufacturing plan	6	Machine useability optimization (availability and energy efficiency)
		7	Flexible and modular hardware	7	Human subjectivity level minimization
		8	Cloud computing	8	Automatic definition of manufacturing plan
		9	Predictive maintenance of hardware and software	9	Automatic definition of technologies and operation sequencing
		10	Excellent computer infrastructure	10	Automatic definition of tools, machines, fixtures etc.
		11	High level of data and connection security	11	Automatic recognition of geometrical features
		12	ERP systems		
		13	Big Data Manipulation		
		14	Connection with outer databases		

## Data Availability

Not applicable.

## References

[1] Misita M., Lapcevic N., Tadic D., Milanovic D.D., Borota-Tisma A. (2016). New Model of Enterprises Resource Planning Implementation Planning Process in Manufacturing Enterprises. Adv. Mech. Eng..

[2] 2019 IEEE Pune Section International Conference (PuneCon): MIT World Peace University, Pune, India, 18–20 December 2019. http://link.library.missouri.edu/portal/2019-IEEE-Pune-Section-International-Conference/b9NeTB0kNxU/.

[3] Essential Implications of the Digital Transformation in Industry 4.0. Web of Science Core Collection. https://www.webofscience.com/wos/woscc/full-record/WOS:000407529700002.

[4] Change Readiness as a Proposed Dimension for Industry 4.0 Readiness Models. Web of Science Core Collection. https://www.webofscience.com/wos/woscc/full-record/WOS:000609424000007.

[5] Kreis A., Hirz M., StadlerKreis S. IEEE Optimized Information Exchange Process between CAD and CAM. Proceedings of the 2018 5th International Conference on Industrial Engineering and Applications (ICIEA) 2018.

[6] Xu X., Wang L., Newman S.T. (2010). Computer-Aided Process Planning-A Critical Review of Recent Developments and Future Trends. Int. J. Comput. Integr. Manuf..

[7] Zhang Y., Luo X., Zhang B., Zhang S. (2017). Semantic Approach to the Automatic Recognition of Machining Features. Int. J. Adv. Manuf. Technol..

[8] Trstenjak M., Ćosić P., Antolić D. (2019). Workpiece Classification Criteria in Automated Process Planning. Teh. Vjesn..

[9] Sunil V.B., Pande S.S. (2008). Automatic Recognition of Features from Freeform Surface CAD Models. Comput.-Aided Des..

[10] Su Y., Chu X., Chen D., Sun X. (2018). A Genetic Algorithm for Operation Sequencing in CAPP Using Edge Selection Based Encoding Strategy. J. Intell. Manuf..

[11] Trstenjak M., Cosic P. (2017). Process Planning in Industry 4.0 Environment. Procedia Manuf..

[12] Królikowski M.A., Krawczyk M.B. Does Metal Additive Manufacturing in Industry 4.0 Reinforce the Role of Substractive Machining?. https://link.springer.com/chapter/10.1007/978-3-030-18715-6_13.

[13] Dayam S., Desai K.A., Kuttolamadom M. (2021). In-Process Dimension Monitoring System for Integration of Legacy Machine Tools into the Industry 4.0 Framework. Smart Sustain. Manuf. Syst..

[14] Moreno A., Velez G., Ardanza A., Barandiaran I., de Infante Á.R., Chopitea R. (2017). Virtualisation Process of a Sheet Metal Punching Machine within the Industry 4.0 Vision. Int. J. Interact. Des. Manuf..

[15] Kurth R., Tehel R., Päßler T., Putz M., Wehmeyer K., Kraft C., Schwarze H. (2019). Forming 4.0: Smart Machine Components Applied as a Hybrid Plain Bearing and a Tool Clamping System. Procedia Manuf..

[16] Akyazi T., Goti A., Oyarbide-Zubillaga A., Alberdi E., Carballedo R., Ibeas R., Garcia-Bringas P. (2020). Skills Requirements for the European Machine Tool Sector Emerging from Its Digitalization. Metals.

[17] Ferreira F., Guerra H. (2018). The Coordinate Measuring Machines, Essential Tools for Quality Control of Dimensional and Geometrical Specifications of Technical Components, in the Context of the Industry 4.0. J. Phys. Conf. Ser..

[18] Singh G., Aggarwal V., Singh S. (2020). Critical Review on Ecological, Economical and Technological Aspects of Minimum Quantity Lubrication towards Sustainable Machining. J. Clean. Prod..

[19] Cañizares E., Valero F.A. (2018). Analyzing the Effects of Applying IoT to a Metal-Mechanical Company. J. Ind. Eng. Manag..

[20] Maier W., Möhring H.C., Werkle K. (2018). Tools 4.0-Intelligence Starts on the Cutting Edge. Procedia Manuf..

[21] Knittel D., Makich H., Nouari M. (2019). Milling Diagnosis Using Artificial Intelligence Approaches. Mech. Ind..

[22] Stentoft J., Adsbøll Wickstrøm K., Philipsen K., Haug A. (2021). Drivers and Barriers for Industry 4.0 Readiness and Practice: Empirical Evidence from Small and Medium-Sized Manufacturers. Prod. Plan. Control..

[23] Industrie 4.0-Readiness-Check. https://www.industrie40-readiness.de/?lang=en.

[24] Industry 4.0 Strategy Consulting Services | BCG. https://www.bcg.com/capabilities/manufacturing/industry-4.0.

[25] Manufacturing’s next Act | McKinsey. https://www.mckinsey.com/business-functions/operations/our-insights/manufacturings-next-act.

[26] Industry 4.0-Self Assessment. https://i40-self-assessment.pwc.de/i40/landing/.

[27] Schumacher A., Erol S., Sihn W. (2016). A Maturity Model for Assessing Industry 4.0 Readiness and Maturity of Manufacturing Enterprises. Procedia CIRP.

[28] Ganzarain J., Ganzarain J., Errasti N. (2016). Three Stage Maturity Model in SME’s toward Industry 4.0. J. Ind. Eng. Manag..

[29] Babi Z., Veža I., Pavić I. Ranking of Enterprises with Regard to Industrial Maturity Level Using AHP and TOPSIS. https://www.bib.irb.hr/830323.

[30] Koska A., Oska A., Goksu N., Erdem M.B., Fettahlioglu H.S. (2017). Measuring the Maturity of a Factory for Industry 4.0. International. J. Acad. Res. Bus. Soc. Sci..

[31] Christian L., Katja B., Thomas S., Sven F. SIMMI 4.0—A Maturity Model for Classifying the Enterprise-Wide It and Software Landscape Focusing on Industry 4.0. Proceedings of the 2016 Federated Conference on Computer Science and Information Systems (FedCSIS).

[32] Godsell J., Agca O., Gibson J., Ignatius J., Davies C.W., Xu O. (2018). An Industry 4 Readiness Assessment Tool. The University of Warwick in association with Crimson & Co, Pinset Masons. https://warwick.ac.uk/fac/sci/wmg/research/scip/reports/final_version_of_i4_report_for_use_on_websites.pdf.

[33] Felch V., Asdecker B., Sucky E. Digitization in Outbound Logistics—Application of an Industry 4.0 Maturity Model for the Delivery Process. https://fis.uni-bamberg.de/handle/uniba/45549.

[34] Kaltenbach F., Marber P., Gosemann C., Bolts T., Kuhn A. Smart Services Maturity Level in Germany. Proceedings of the 2018 IEEE International Conference on Engineering, Technology and Innovation (ICE/ITMC).

[35] Canetta L., Barni A., Montini E. Development of a Digitalization Maturity Model for the Manufacturing Sector. Proceedings of the 2018 IEEE International Conference on Engineering, Technology and Innovation (ICE/ITMC).

[36] Basl J., Doucek P. (2019). A Metamodel for Evaluating Enterprise Readiness in the Context of Industry 4.0. Information.

[37] Trotta D., Garengo P. Assessing Industry 4.0 Maturity: An Essential Scale for SMEs. Proceedings of the 2019 8th International Conference on Industrial Technology and Management (ICITM).

[38] Gracel J., Łebkowski P. (2018). The Concept of Industry 4.0 Related Manufacturing Technology Maturity Model (Manutech Maturity Model, MTMM). Decis. Mak. Manuf. Serv..

[39] Oleśków-Szłapka J., Stachowiak A. (2019). The Framework of Logistics 4.0 Maturity Model. Adv. Intell. Syst. Comput..

[40] Maisiri W., Van Dyk L. (2019). Industry 4.0 Readiness Assessment for South African Industries. S. Afr. J. Ind. Eng..

[41] SIRI Workshop on Industry 4.0 Maturity | Digital Enterprise | Siemens Global. https://new.siemens.com/global/en/company/topic-areas/digital-enterprise/digitalization-check.html.

[42] Machado C.G., Winroth M., Carlsson D., Almström P., Centerholt V., Hallin M. (2019). Industry 4.0 Readiness in Manufacturing Companies: Challenges and Enablers towards Increased Digitalization. Procedia CIRP.

[43] Vrchota J., Pech M. (2019). Readiness of Enterprises in Czech Republic to Implement Industry 4.0: Index of Industry 4.0. Appl. Sci..

[44] Pirola F., Cimini C., Pinto R. (2020). Digital Readiness Assessment of Italian SMEs: A Case-Study Research. J. Manuf. Technol. Manag..

[45] Lucato W.C., Pacchini A.P.T., Facchini F., Mummolo G. (2019). Model to Evaluate the Industry 4.0 Readiness Degree in Industrial Companies. IFAC-Pap..

[46] Santos R.C., Martinho J.L. (2019). An Industry 4.0 Maturity Model Proposal. J. Manuf. Technol. Manag..

[47] De Bruin T., Freeze R., Kulkarni U., Rosemann M. Understanding the Main Phases of Developing a Maturity Assessment Model. https://eprints.qut.edu.au/25152/.

[48] Antony J., Sony M., McDermott O. Conceptualizing Industry 4.0 Readiness Model Dimensions: An Exploratory Sequential Mixed-Method Study. https://www.researchgate.net/publication/27482282_Understanding_the_Main_Phases_of_Developing_a_Maturity_Assessment_Model.

[49] Wagire A.A., Joshi R., Rathore A.P.S., Jain R. (2020). Development of Maturity Model for Assessing the Implementation of Industry 4.0. Learning from Theory and Practice. Prod. Plan. Control..

[50] Kruger S., Steyn A.A. (2020). A Conceptual Model of Entrepreneurial Competencies Needed to Utilise Technologies of Industry 4.0. Int. J. Entrep. Innov..

[51] Sriram R.M., Vinodh S. (2021). Analysis of Readiness Factors for Industry 4.0 Implementation in SMEs Using COPRAS. Int. J. Qual. Reliab. Manag..

[52] Caiado R.G.G., Scavarda L.F., Gavião L.O., Ivson P., de Mattos Nascimento D.L., Garza-Reyes J.A. (2021). A Fuzzy Rule-Based Industry 4.0 Maturity Model for Operations and Supply Chain Management. Int. J. Prod. Econ..

[53] Industry 4.0 Maturity Model—Mirroring Today to Sprint into the Future | Supply Chain Management Blog. https://www.capgemini.com/ch-en/2018/02/industry-4-0-maturity-model-mirroring-today-to-sprint-into-the-future/.

[54] Popkova E.G., Egorova E.N., Popova E., Pozdnyakova U.A. (2019). The Model of State Management of Economy on the Basis of the Internet of Things. Stud. Comput. Intell..

[55] Brozzi R., D’Amico R.D., Pasetti Monizza G., Marcher C., Riedl M., Matt D. (2018). Design of Self-Assessment Tools to Measure Industry 4.0 Readiness. A Methodological Approach for Craftsmanship SMEs. IFIP Adv. Inf. Commun. Technol..

[56] Sternad M., Lerher T., Gajšek B. Maturity Levels For Logistics 4.0 Based On New’s Industry 4.0 Maturity Model. https://ideas.repec.org/a/osi/bulimm/v18y2018p695-708.html.

[57] Santos K., Rocha Loures E.F., Junior O., Santos E. Product Lifecycle Management Maturity Models in Industry 4.0. IFIP Advances in Information and Communication Technology. https://hal.inria.fr/hal-02075595.

[58] Staufen A.G. (2018). German Industry 4.0 Index. A study from Staufen AG and Staufen Digital Neonex GmbH. https://www.staufen.ag/fileadmin/HQ/02-Company/05-Media/2-Studies/STAUFEN.-Study-Industry-4.0-Index-2018-Web-DE-en.pdf.

[59] Gaps in Industry 4.0 Readiness Contribute to Industrie 4.0 Maturity Index. https://www.i-scoop.eu/industry-4-0/gaps-industrie-4-0-maturity-index/.

[60] Ratnasingam J., Latib H.A., Yi L.Y., Liat L.C., Khoo A. (2019). Extent of Automation and the Readiness for Industry 4.0 among Malaysian Furniture Manufacturers. BioResources.

[61] De Carolis A., Macchi M., Negri E., Terzi S. A Maturity Model for Assessing the Digital Readiness of Manufacturing Companies. https://link.springer.com/chapter/10.1007/978-3-319-66923-6_2.

[62] A Strategist’s Guide to Industry 4.0. https://www.strategy-business.com/article/A-Strategists-Guide-to-Industry-4.0.

[63] (2016). McKinsey&Company, Industry 4.0 at McKinsey’s Model Factories Get Ready for the Disruptive Wave. http://sf-eu.net/wp-content/uploads/2016/08/mckinsey-2016-industry-4.0-at-mckinseys-model-factories-en.pdf.

[64] Readiness for the Future of Production Report 2018–Kearney. https://www.kearney.com/operations-performance-transformation/article?/a/readiness-for-the-future-of-production-report-2018.

[65] Berger Strategy Consultants R., Blanchet M., Rinn T., de Thieulloy G., von Thaden G. (2014). Industry 4.0 The New Industrial Revolution—How Europe Will Succeed. http://www.iberglobal.com/files/Roland_Berger_Industry.pdf.

[66] Industrie 4.0 Maturity Index. Managing the Digital Transformation of Companies—UPDATE 2020-Acatech-National Academy of Science and Engineering. https://en.acatech.de/publication/industrie-4-0-maturity-index-update-2020/.

[67] Trstenjak M., Opetuk T., Cajner H., Tosanovic N. (2020). Process Planning in Industry 4.0—Current State, Potential and Management of Transformation. Sustainability.

[68] Saaty R.W. (1987). The Analytic Hierarchy Process—What It Is and How It Is Used. Math. Model..

